# Frozen-section examination in the management of paediatric testicular lesions

**DOI:** 10.1007/s00383-021-04870-w

**Published:** 2021-03-13

**Authors:** E. O’Connor, C. Roy, S. Annavarapu, H. O. Gabra

**Affiliations:** 1 Department of Paediatric Surgery, Great North Children’s Hospital, Royal Victoria Infirmary, Newcastle upon Tyne, NE1 4LP UK; 2 Department of Cellular Pathology, Great North Children’s Hospital, Royal Victoria Infirmary, Newcastle upon Tyne, NE1 4LP UK

**Keywords:** Frozen-section examination, Testis-sparing surgery, Testicular sparing, Testicular and para-testicular lesions, Paediatric patients

## Abstract

**Purpose:**

Paediatric testicular and para-testicular lesions have traditionally been managed according to adult protocols. Testis-sparing surgery (TSS) has gained popularity as it has become apparent benign lesions predominate in childhood. Frozen-section examination (FSE) for intra-operative diagnosis has been extensively utilised in adults, though its use in paediatric practice remains limited. We reviewed our experience of FSE in paediatric patients with an aim to identify the utility and efficacy of this tool in the management of testicular and para-testicular pathology.

**Methods:**

A retrospective, single-centre review of paediatric patients who underwent intra-operative FSE for a range of testicular and para-testicular lesions was performed. FSE results were compared to final pathology. TSS was performed if appropriate, and was utilised in adolescent patients, and in lesions with a diameter greater than 20 mm.

**Results:**

Nine males underwent FSE from 2013 to 2020. Median age at surgery was 9 years (range 1–15). Eight (89%) patients had benign pathology. FSE result correlated with the final pathological examination in 100% of cases. FSE facilitated TSS in 7/9 cases.

**Conclusion:**

FSE has 100% diagnostic accuracy for paediatric testicular and para-testicular pathology. We would recommend all lesions be evaluated by FSE to guide intra-operative decision making and facilitate TSS in appropriate cases.

## Introduction

Traditionally paediatric testicular tumours have been managed according to adult protocols, with radical inguinal orchidectomy [1, 2]. As the true incidence of malignant testicular pathology in childhood is much lower than previously thought [3–5], testis sparing surgery (TSS) has become a desirable and viable option. Despite extensive data from adult literature showing the efficacy of frozen-section analysis in the diagnosis of benign and malignant testicular lesions [6–8], the use of frozen-section examination (FSE) as a diagnostic tool in paediatric testicular and para-testicular tumours remains limited. Most reports are small case series focussing on TSS [9–11].

The aim of this study was to review our institutional experience regarding the role of intra-operative FSE in the diagnosis and management of testicular and para-testicular lesions in paediatric patients.

## Methods

A retrospective, single-centre review of a cohort of patients who underwent intra-operative FSE for testicular and para-testicular lesions between 2013 and 2020 was undertaken. Data analysed included demographics, pre-operative laboratory investigations, radiological imaging, operative findings, FSE results and final pathology.

All patient management plans were discussed with, and approved by the local oncology multidisciplinary team. Surgery was performed by the senior author (HG) in all cases. The lesion was approached via a standard inguinal incision. The cord structures were identified, mobilised and isolated with a soft clamp. The testis was delivered out of the wound and inspected in relation to pre-operative imaging. The tunica albuginea was incised and an excision biopsy of the lesion was performed and sent for intra-operative FSE.

Samples were placed on a metal disc and embedded in a gel medium—Optimal Cutting Temperature compound (OCT). They were frozen rapidly to − 20 °C so that the tissue hardened. They were then secured on a chuck and 6–8 μm sections were cut using a cryostat microtome. The cryosections were fixed on glass slides, dried for 15 s and rapidly stained with Haematoxylin and Eosin stain (H&E). FSE was performed by three senior pathologists. The average time taken for analysis was 20 min (although this was not formally measured).

If the lesion was confirmed benign and suitable for TSS, the tunica was closed with absorbable sutures and the testis returned to the scrotum. If the lesion was identified as malignant, a radical orchidectomy was performed. Patients received follow-up with surgical, oncology and endocrine teams as appropriate.

Ethical approval was not required for this study, though institutional approval for a quality improvement project was granted. Data were summarised with counts and frequencies for categorical variables and percentages were employed to describe relative proportions.

## Results

A total of 12 patients were operated on for testicular or para-testicular lesions during the study period. Three patients were excluded because they underwent orchidectomy for suspected malignancy without undergoing FSE.

Nine boys (median age 9 years, range 1–15) underwent FSE of testicular and para-testicular lesions. Five patients presented with a painless mass and four had a lump associated with intermittent or progressive pain. Five lesions were in the right hemi-scrotum, and four were in the left. Four were testicular lesions and five para-testicular. The mean lesion diameter was 25 mm (range 4–50 mm) (Table 1).

**Table 1:** Summary of cases, biochemical and radiological findings, frozen section result and final histopathology

N	Age (years)	Presentation	Biochemistry	Ultrasound conclusion	Frozen section	Size (width; mm)	Final pathological diagnosis	TSS	Duration of surgery (min)	Follow-up (months)
1	1	Right testicular lump increasing in size for 6 months	Not available	Unable to exclude malignancy	Benign	30	Para-testicular fibrous hamartoma of infancy	Yes	No data available	1
2	15	Painless large left testicular and para-testicular mass	AFP/HCG/LDH negative	?malignant	Benign	50	Cystic dysplasia of rete testis	No	59	33
3	8	Short history of painless hard right testicle	LDH 266, AFP/HCG negative	?malignant	Malignant	42	Para-testicular rhabdomyosarcoma	No	90	37
4	11	Intermittent right testicular pain and a palpable lesion	Not available	Unable to exclude malignancy	Benign	4	Tubular ectasia of rete testis and epididymis	Yes	96	2
5	3	Right testicular mass. Inguinal and cervical lymphadenopathy	LDH 1036, AFP and HCG negative	Suspicious for malignancy	Benign	9	Epidermoid cyst	Yes	55	36
6	15	Left testicular lump, intermittently tender and slowly growing over 2 months	AFP/HCG negative	Likely benign ?epidermoid cyst	Benign	16	Epidermoid cyst	Yes	107	20
7	15	Painless left testicular lump in a patient with congenital adrenal hyperplasia	AFP/BHCG negative	Suspicious for malignancy	Benign	10	Testicular adrenal rest tumour (TART)	Yes	80	62
8	9	8 months of painful left testis and overlying cellulitis	AFP/HCG/LDH negative	Likely benign	Benign	30	Haematocele	Yes	71	0
9	7	2 months of intermittent right scrotal pain and swelling	AFP/HCG/LDH negative	Likely benign? lymphatic malformation	Benign	38	Lympho-vascular malformation	Yes	82	4

*TSS* testis-sparing surgery, *FS* frozen section, *AFP* alpha-fetoprotein, *HCG* human chorionic gonadotropin, *LDH* lactate dehydrogenase, *US* ultrasound, *Min* Minutes

### Biochemistry

Seven patients had pre-operative serum tumour markers available. Two patients, one with a rhabdomyosarcoma and one with a benign epidermoid cyst, had a raised lactate dehydrogenase (LDH). α-Feto protein (AFP) and β-HCG were negative in all cases (Table 1).

### Imaging

Scrotal ultrasound (US) was performed in all cases. When looking at final pathology, US had correctly identified three benign lesions. However, pre-operative ultrasound was unable to exclude malignancy in six suspicious lesions. Five of these inconclusive lesions were shown to be benign on FSE. (Table 1).

### Frozen-section examination (FSE)

Intra-operative frozen section was performed for nine testicular and para-testicular lesions. The result correlated with the final pathology in all cases, giving a 100% diagnostic accuracy.

**Fig. 1.**
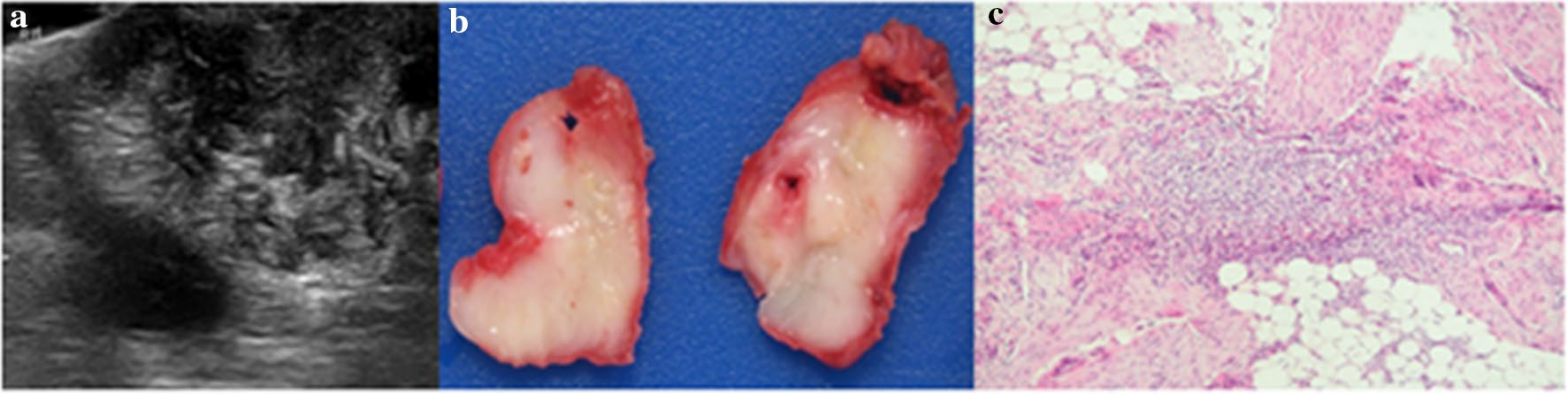
11-month male presented with a firm right para-testicular mass. **a** Ultrasound image shows a heterogenous area of mixed echogenicity 3 × 2 cm, causing mass-effect and displacing the testis superiorly. **b** Intra-operative frozen section confirmed a benign lesion. Histology showed the typical tri-phasic appearance of fibrous hamartoma of infancy. **c** Pathology. A testis-sparing resection. Greyish-white mass with homogeneous areas and no haemorrhage or necrosis. Final diagnosis: Fibrous hamartoma of infancy

**Fig. 2:**
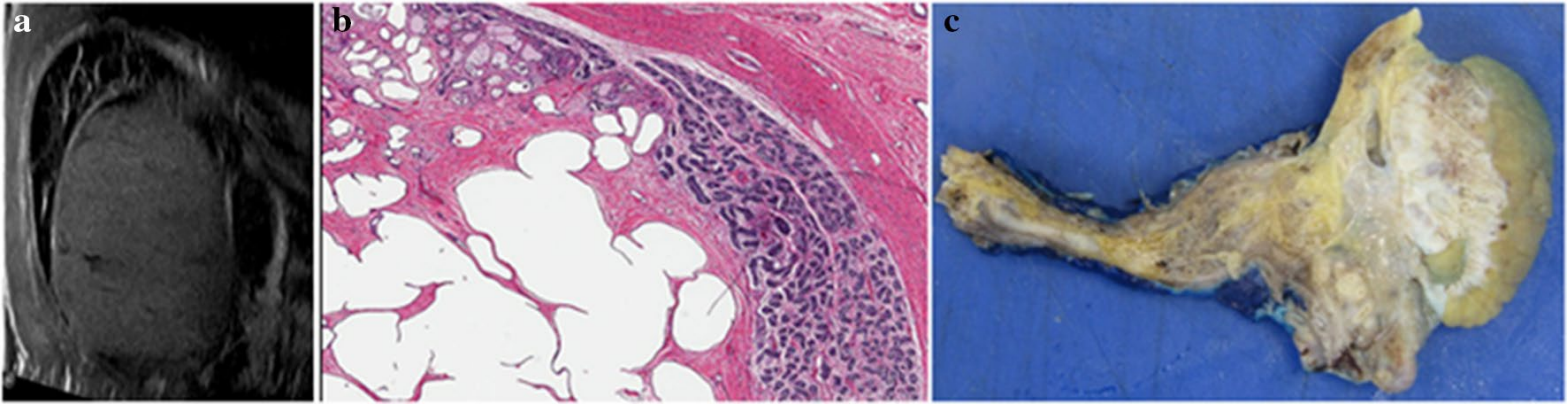
Large complex left para-testicular mass in 14-year-old male. **a** Ultrasound: distortion of the upper pole with a focal hypoechoic lesion with peripheral blood flow. Adjacent, but separate to this mass is an ill-defined heterogeneous area of abnormality which appears infiltrative into the normal parenchyma. **b** Intra-operative frozen section confirmed benign pathology. Benign multi-cystic lesion comprising variable sized cysts compressing the native testis and mediastinal rete testis. **c** Pathology—cystic dysplasia of the rete testis. Orchidectomy was performed as the lesion was so large and rest of the testicular tissue was atrophic. Final pathology: cystic dysplasia of the rete testis

**Fig. 3.**
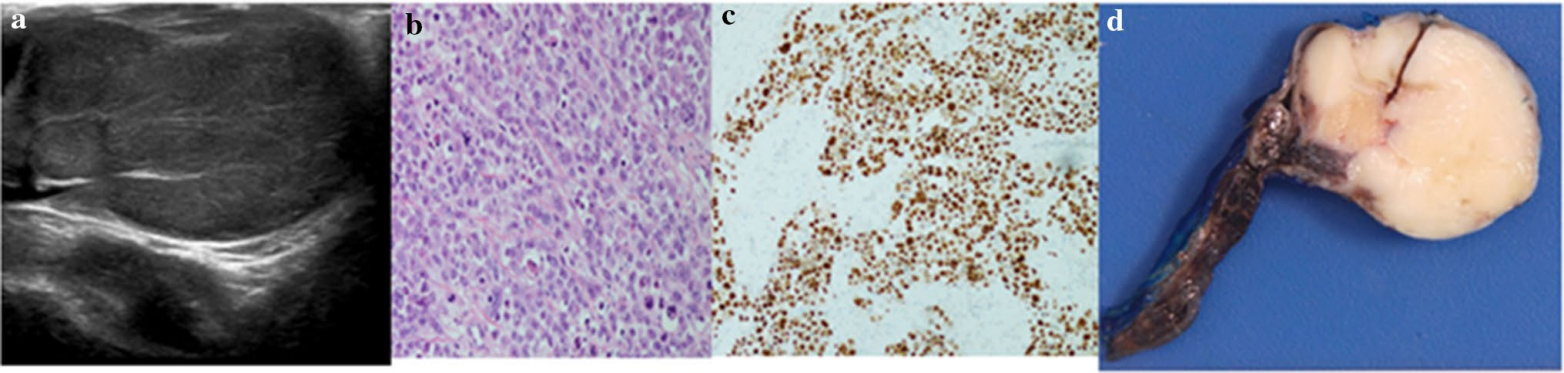
8-year male with a para-testicular mass. **a** Ultrasound shows an unusual appearance of the right testis which is grossly oedematous and of heterogeneous reflectivity, features highly suspicious for malignancy. **b** Histology: typical morphology of alveolar rhabdomyosarcoma. **c** Myogenin staining shows nuclear positivity, diagnostic of rhabdomyosarcoma. **d** Pathology specimen. Radical orchidectomy for para-testicular rhabdomyosarcoma. Final pathology: para-testicular rhabdomyosarcoma.

Benign lesions were identified in eight patients (89%). One para-testicular fibrous hamartoma of infancy (Fig. 1), one cystic dysplasia of the rete testis (Fig. 2), one tubular ectasia of the rete testis, two epidermoid cysts, one testicular adrenal rest tumour (TART), one haematocele and one lymphatic malformation (Table 1). One malignant lesion was identified—a para-testicular rhabdomyosarcoma (Fig. 3).

### Testis-sparing surgery (TSS)

TSS was successfully performed in seven patients following FSE. A size cut-off of 20 mm did not preclude TSS; it was performed in lesions measuring up to 38 mm (Table 1). TSS was successfully performed in two adolescent patients, aged 15 years, with benign pathology (epidermoid cyst and TART).

Orchidectomy was performed in two patients post-FSE. One had malignancy confirmed on FSE, with final diagnosis of a para-testicular rhabdomyosarcoma. The other had benign pathology on FSE, but orchidectomy was deemed necessary as the lesion took up the entire testis. Final pathology showed cystic dysplasia of the rete testis.

Median operative time was 81 min (range 55–107). This included time for FSE.

Median follow-up for all patients was 26.5 months (range 1–62).

## Discussion

Individual surgeon series and multi-centre reports of testicular and para-testicular pathology have shown benign lesions are more common in younger boys (up to 74%) compared to their adolescent and adult counterparts [2–5]. Our observations correlate with this finding, as 89% of lesions in our cohort were benign. TSS has, therefore, become an attractive option to avoid unnecessary orchidectomy in this age group [2, 9, 10]. Preserving testicular tissue has significant cosmetic and psychological advantages for children and important implications for future fertility [12, 13]. Furthermore, orchidectomy has also been shown to be associated with osteopenia [10, 14].

FSE for testicular lesions has been shown to have high diagnostic accuracy in adults. In 2002, Tokuc et al. reviewed 26 cases and found frozen section correctly identified all malignant and benign testicular masses [6]. Other large patient series have shown FSE has a sensitivity of 95–100% and a specificity of 100% for adult pathology [7, 8, 15–17]. Despite FSE being widely accepted in the management of adult testicular and para-testicular pathology, there remains limited published data from paediatric practice. Most are small case series focussing on TSS [5, 9, 10, 18]. Radford et al. remark that despite FSE showing almost 100% specificity, it is not always performed in childhood testicular lesions [11]. This may be due to persistence of the belief that the majority of paediatric testicular lesions are malignant and should be treated accordingly. In addition, the use of FSE relies on the presence of a pathologist experienced in this technique, which may not be readily available to all. There is also a reluctance of the pathologists to use FSE for diagnosis, as the quality is regarded as inferior to the routine formalin-fixed paraffin-embedded tissue processing (personal communications).

FSE has been utilised in paediatric patients. Emre et al. described four cases where FSE correctly confirmed final pathology for Leydig cell tumours [18], and Zu’bi et al. report 85% accuracy when FSE result was compared to final histology reports for a cohort of nine patients, although FSE was only performed in 88% of cases [9].

In 2019, Caldwell et al. showed good correlation between frozen section and final pathology for 24 pre- and post-pubertal boys. They found frozen section more accurately predicted pathology than tumour size, and recommended the use of FSE for the intra-operative diagnosis of benign or malignant lesions [10]. Our findings support Caldwell et al. We have shown FSE correctly identified benign and malignant pathology in all cases, with a 100% diagnostic accuracy. In our cohort, a tumour size cut-off of 20 mm did not preclude TSS, it was performed in three benign lesions measuring greater than 20 mm (Table 1).

Shukla et al*.* reported their experience with TSS for testicular teratoma in childhood. They found FSE had a good correlation with final pathology. As their experience progressed, they developed more reliance on serum tumour markers, US and intra-operative appearance, and subsequently, for most cystic lesions, they did not perform FSE [5]. Despite the excellence of paediatric US, in our cohort, US could not exclude malignancy in six patients. FSE showed five of these suspicious lesions were benign, enabling TSS in four. US can be a helpful diagnostic tool, but to obtain a greater degree of certainty, we would recommend FSE be performed on all suspicious lesions to confirm if they are benign or malignant.

Although TSS has been shown to be safe in pre-pubertal boys, adolescent males have still been treated according to adult protocols, as they have a much higher incidence of malignancy [10]. In our cohort, there were three males aged 15 years. FSE correctly identified benign pathology in all cases (cystic dysplasia of the rete testis, epidermoid cyst and TART), and facilitated TSS in two of them, showing FSE and TSS can still be a viable option in adolescent males. It has been suggested that FSE of surrounding tissue should be performed in adolescents presenting with teratomas, and if there is evidence of pubertal change, the patient should undergo orchidectomy, as adolescent teratomas tend to be more aggressive than in their paediatric counterparts [5].

Our results showing FSE has a 100% diagnostic accuracy in paediatric and adolescent testicular and para-testicular lesions are promising, as FSE can facilitate TSS and avoid orchidectomy in young patients. However, this study is limited by its small sample size, and consequently, our conclusions require further validation from a larger cohort analysis.

Many other series looking at TSS in the paediatric population have small patient numbers [2, 5, 9, 10, 18], and our small cohort may simply reflect the rarity of the condition. As suggested by Radford et al. (11), multi-centre collaboration would be a way to increase our sample size and is something to be considered for the future, looking at both TSS, as they suggest, but also at the accessibility and efficacy of FSE in different centres. Three adolescents successfully underwent FSE, and two went on to have TSS, but with only three cases, much more work is required before we can draw definite conclusions in this higher risk patient population.

This was a retrospective review, and is, therefore, subject to the usual biases associated with this type of study. The nine patients who underwent FSE will have been affected by selection bias and may not be representative of the general population. Currently, there is only one surgeon who performs TSS in our centre. The use of FSE and subsequent TSS will have depended on the availability of the surgeon and pathologists experienced in FSE, which may not be accessible to all.

Historically, if there was a suspicious testicular or para-testicular lesion on clinical examination and imaging, FSE may not have been performed, as it would be assumed these patients would be undergoing orchidectomy (as was the case for the three patients excluded from this series). However, we have shown pre-operative imaging is not always conclusive, and in six suspicious lesions on pre-operative ultrasound, four patients were subsequently able to undergo TSS due to FSE showing benign pathology. As such, we advocate using FSE on all suspicious lesions to avoid unnecessary orchidectomy. In the future, we should identify clinical and imaging selection criteria as to which patients would be suitable for FSE, to give all patients the option of TSS when appropriate.

In conclusion, although our sample size is small, we have shown FSE results correlate with the final pathology in 100% of cases. Due to this high diagnostic accuracy, we would recommend FSE be used in all paediatric patients to diagnose suspicious testicular and para-testicular pathology. Further work including meta-analysis and multi-centre collaboration would give a larger cohort of patients to validate our conclusions about the benefits of using FSE to help avoid unnecessary orchidectomy in paediatric and adolescent patients.
